# MTHFD1L-Mediated Redox Homeostasis Promotes Tumor Progression in Tongue Squamous Cell Carcinoma

**DOI:** 10.3389/fonc.2019.01278

**Published:** 2019-12-05

**Authors:** Hao Li, Xiaoyan Fu, Fan Yao, Tian Tian, Chunyang Wang, Ankui Yang

**Affiliations:** ^1^State Key Laboratory of Oncology in South China, Collaborative Innovation Center for Cancer Medicine, Sun Yat-sen University Cancer Center, Guangzhou, China; ^2^Department of Head and Neck Surgery, Sun Yat-sen University Cancer Center, Guangzhou, China; ^3^Guanghua School of Stomatology, Hospital of Stomatology, Sun Yat-sen University, Guangzhou, China

**Keywords:** tongue squamous cell carcinoma, MTHFD1L expression, anti-oxidant activity, tumorigenesis, mTORC1

## Abstract

**Background:** Routine changes in cell metabolism can drive tumor development, as the cellular program develops to promote glycolysis and redox homeostasis during tumor progression; however, the associated mechanisms in tongue squamous cell carcinoma (TSCC) remain unclear.

**Methods:** We investigated methylenetetrahydrofolate dehydrogenase 1-like (MTHFD1L) expression, its clinical relevance, redox modification, and molecular mechanisms using TSCC cells and tissues. The anti-tumor effects of MTHFD1L knockdown on TSCC tumorigenesis were evaluated *in vitro* and *in vivo*. Kaplan-Meier curves and the log-rank test were used to analyze disease-free survival and overall survival.

**Results:** TSCC patients with high expression levels of MTHFD1L had shorter overall survival (*P* < 0.05) and disease-free survival (*P* < 0.05). Knockdown of MTHFD1L reduced nicotinamide adenine dinucleotide phosphate (NADPH) levels and increased reactive oxygen species (ROS), which accelerated cell death under oxidative stress, such as hypoxia or glucose deprivation. Additionally, inhibition of MTHFD1L suppressed TSCC cell growth and delayed the cell cycle, including in xenograft experiments.

**Conclusions:** MTHFD1L confers redox homeostasis and promotes TSCC cell growth, which provides a great opportunity to study tumor metabolism in head and neck cancer. The mTORC1-4EBP1-eIF4E axis may affect the expression of MTHFD1L in TSCC. Inhibition of the expression of MTHFD1L may be an actionable and effective therapeutic target in TSCC.

## Introduction

As a subset of oral cancer, tongue squamous cell carcinoma (TSCC) constitutes more than half of oral SCCs in China and is characterized by its high incidence and poor prognosis (1–3). Despite advances in medical treatments, there are still many TSCC patients who are ineligible for surgical therapy and who experience recurrence, metastasis and radiochemotherapy resistance, making TSCC very difficult for doctors and miserable for patients. Therefore, the identification of a reliable prognostic biomarker or promising therapeutic target for TSCC is a pressing task. Changes in cell metabolism are known to drive tumor development. Numerous studies have confirmed that metabolic reprogramming occurs in tumorigenesis and in development (4, 5). Our recent studies investigated the roles of metabolic reprogramming in promoting glycolysis and redox hemostasis (6–8); however, the regulation of NADP metabolism in TSCC remains unclear and is of interest.

When angiogenesis lags around tumor cells, ischemic and hypoxic microenvironments are formed in local tumor tissues, and a large amount of reactive oxygen species (ROS) are produced. ROS are molecules such as O_2_•−, H_2_O_2_, HO_2_•, and HO• that contain one or more unpaired electrons in their orbital that are can participate in a considerable amount of oxidation reactions (9). ROS are generated from several different sources, including NADPH oxidases, mitochondria, cytochrome P450, and xanthine oxidase (10–13). In cells, high levels of ROS cause irreversible damage to cellular components, leading to cell-cycle arrest and apoptosis (14). Thus, to survive oxidative stress, cancer cells need increased anti-oxidant capacity (15, 16). The folate cycle plays a central role in cell metabolism (17) and produces metabolites that are critical for cell growth, including nucleotides and the major cellular anti-oxidant source NADPH (18, 19). One study demonstrated that the most overexpressed NADPH-generating enzyme in the folate cycle, methylene tetrahydrofolate dehydrogenase 2 (MTHFD2), was associated with colorectal cancer progression (20). MTHFD1L catalyzes 10-formyl-THF to formate, which is the final step in the flow of 1C units from mitochondria to cytoplasm (21), acting important roles in embryonic development. As well, MTHFD1L encodes formyltetrahydrofolate synthetase and is involved in the synthesis of tetrahydrofolate (THF) in the mitochondria, playing critical roles in folate cycle maintenance (18, 22, 23). It has been reported that MTHFD1L overexpressed in many types of cancers, and promoted tumorigenesis and tumor progression (18, 24–27).

The clinical relevance, function, and underlying regulatory mechanisms of MTHFD1L were investigated in this study. Here, we hypothesized that MTHFD1L plays an important role in TSCC cell survival during oxidative stress, resulting in TSCC progression. We expect MTHFD1L to be a new diagnostic and therapeutic target for TSCC.

## Materials and Methods

### Ethics Statement

We obtained human samples from Sun Yat-sen University Cancer Center; the tumor specimens used for the research were approved by the Ethics Committee of Sun Yat-sen University Cancer Center, and the TSCC patients in the study signed consent forms. All methods were performed according to the relevant guidelines.

### Patients and Tumor Tissue Samples

In this study, we collected 106 TSCC pathological specimens at diagnosis from the Head and Neck Department at Sun Yat-sen University Cancer Center between September 2005 and February 2009. In this study, all of the patients were first diagnosed with TSCC at Sun Yat-sen University Cancer Center according to the histological criteria of the World Health Organization (WHO). The patients' clinicopathological records used the American Joint Committee on Cancer (AJCC) Cancer Staging Manual (Seventh Edition). The clinicopathological features of the 106 patients are shown in Table 1.

**Table 1:** The clinicopathological features of MTHFD1L in TSCC.

**Variable**	**MTHFD1L**			***P***
	**Low**	**Medium**	**High**	
**AGE**				
<60	18	53	3	**0.024**
≥60	12	15	5	
**GENDER**				
Male	14	42	6	0.232
Female	16	26	2	
**T CLASSIFICATION**				
I	26	44	1	
II	3	19	1	**<0.001**
III	0	2	2	
IV	1	3	4	
**N CLASSIFICATION**				
N0	28	50	3	**0.003**
Nx	2	18	5	
M classification				
M0	30	67	8	0.754
M1	0	1	0	
**TNM STAGING**				
I	25	39	1	
II	3	11	1	**0.001**
III	0	8	1	
IV	2	10	5	
**DIFFERENTIATION STAGE**				
I	24	45	4	
II	5	19	3	0.476
III	1	4	1	
**RECURRENCE**				
Yes	23	53	5	0.622
No	7	15	3	

*The relation between the MTHFD1L expression and the clinicopathologic characteristics of TSCC*.

*Bold means the p value is less than 0.05, which is statistically significant*.

### Database

**Figure 1:**
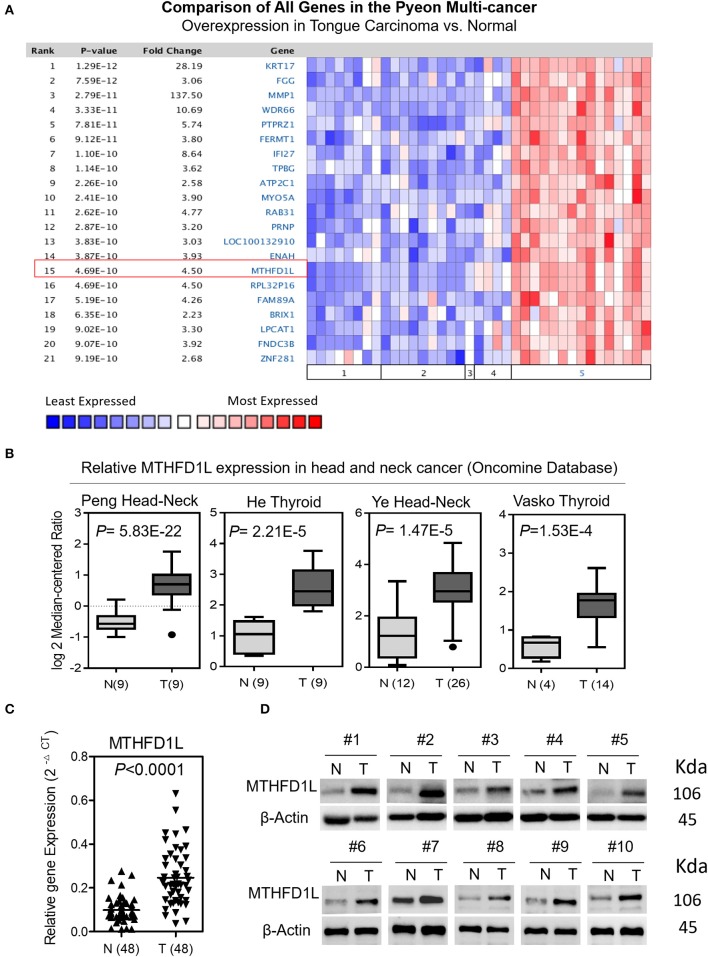
The expression of methylenetetrahydrofolate dehydrogenase 1-like (MTHFD1L) in TSCC. **(A)** Expression of various genes in the Oncomine TSCC database (1. Cervix Uteri; 2. Oral Cavity; 3. Palate; 4. Tonsil; 5. Tongue Carcinoma). **(B)** MTHFD1L expression in multiple Head and Neck cancers microarray data sets is available from the Oncomine database. **(C)** Real time quantitative polymerase chain reaction (qPCR) analysis of the MTHFD1L mRNA expression in 48 paired TSCC tissues. **(D)** Western blotting analysis of the MTHFD1L protein level in TSCC tissues and paired ANTs.

We screened paired TSCC samples (Cancer vs. Normal Analysis) from the Oncomine microarray database (http://www.oncomine.org). MTHFD1L was overexpressed in tongue carcinoma (Figure 1A); then, we used “MTHFD1L” as a keyword in the Oncomine search with “Cancer vs. Normal Analysis” as the primary filter and “Head and Neck cancer” as the cancer type. The mRNA levels of MTHFD1L were upregulated in multiple data sets, including those of Peng, He, Ye, and Vasko. The MTHFD1L expression data were log-transformed and median-centered for each array, and the standard deviation (SD) was normalized to one for each array.

### Gene Set Enrichment Analysis (GSEA)

GSEA was performed using normalized data by GSEA v3.0 tool (http://software.broadinstitute.org/gsea/index.jsp) (28, 29). To explore the differences in potential biological functions in the low- and high-expression sets of MTHFD1L gene, GSEA was used using the Molecular Signatures Database (MSigDB) of Hallmark gene sets (h.all.v6.1.symbols).

### Cell Lines and Cell Culture

A normal tongue epithelial cell line, the Normal Oral Keratinocyte (NOK) cell line, was kindly gifted by the Hospital of Stomatology at Sun Yat-sen University, and the human TSCC cell lines (SCC-9, SCC-15, SCC-25, CAL-27, and Tca8113) were purchased from the Shanghai Institute of Biochemistry and Cell Biology, Cell Bank/Stem Cell Technology platform (CAS). The NOK cell line was cultured in Oral Keratinocyte Medium-basal (ScienCell, SC-2611-b). The human TSCC cell lines (SCC-9, SCC-15, and SCC-25) and CAL-27 were cultured in a mixture of DMEM (Invitrogen) and Ham's F12 medium.The Tca8113 cell line was grown in RPMI-1640 (Invitrogen). The media listed above was supplemented with 10% fatal bovine serum (FBS) (Gibco), 100 μg/mL penicillin/streptomycin (Gibco) and other needed nutrients. All cells were cultured in a humidified incubator with 5% CO_2_ at 37°C.

### shRNA and Stable Cell Lines

To knockdown the MTHFD1L gene expression, TSCC cell lines (CAL-27, SCC-15) were transfected with specific short hairpin MTHFD1L RNAs (shRNAs, 5′-GGCCAAAGCTGTAATTGAACTTCT-3′ and 5′-GCTCTGTATAATCGGCTGGTTC-3′); the shRNAs were purchased from GenePharma Co., Ltd. (Suzhou, China).

We used established stable cell lines from the CAL-27 and SCC-15 cell lines by selection with 3 μg.mL^−1^ puromycin for 3 weeks. We purchased adenoviruses from GenePharma Co., Ltd.

### Antibodies and Western Blot Analysis

We used 10% SDS-PAGE gels to separate equal amounts of protein and transferred the proteins onto polyvinylidene fluoride (PVDF) membranes for detection. The membranes were then sequentially incubated with specific anti-bodies at 4°C overnight; the membranes were then incubated with secondary anti-bodies at room temperature for 1 h the protein bands were detected using enhanced chemiluminescence. Anti-MTHFD1L, anti-MTHFD2 were purchased from Abcam, anti p-S6RP were purchased from Cell Signaling Technology, and anti-β-Actin was purchased from Proteintech (Wuhan, China).

### RNA Extraction and Quantitative RT-PCR (qRT-PCR)

We extracted total RNA from the indicated cells using a RaPure Total RNA Micro Kit (Magen, Guangzhou, China). Then, extracted RNA was reverse transcribed with a ReverTraAce qPCR RT Master Mix kit (ToYoBo, Shanghai, China). The qRT-PCR primers for MTHFD1L (5′- CAACATCAAGTGCCGAGCTT-3′; 5′- AAGAGGAACACCAGCCGTTA−3′), GAPDH (5′- TCGGAGTCAACGGATTTGGT−3′; 5′- TTCCCGTTCTCAGCCTTGAC−3′) were purchased from Invitrogen (Shanghai); qRT-PCR was performed with the SYBR Green Real-time PCR Master Mix (ToYoBo, Shanghai, China). The quantification method was -ddCt method.

### Cell Colony Formation

Cells with MTHFD1L knockdown were seeded into six-well plates (500 cells/well) and were incubated in a humidified 5% CO_2_ incubator at 37°C for 14 days. We used formalin to fix the cells for 10 min and then stained the cells with crystal violet for 15 min. Next, we captured images of the clones and counted the number of clones (colonies with >50 cells were counted) using Image-Pro Plus 6.0 software.

### Cell Proliferation

We performed MTS assay (Promega Biotech Co., Ltd., Madison, WI, USA) to determine the cell viability. We seeded cells into 96-well plates (4,000 cells/well), cultured them overnight, then transfected them with the MTHFD1L shRNA or a negative control. Cell viability was analyzed 1 week after transfection.

### ROS, NADPH and GSH Measurement, Cell Apoptosis and Cell Cycle Analysis

First, cells were cultured in glucose-deprived medium and were incubated with 10 mmol/L 2, 7-dichlorodihydrofluorescein diacetate (H2-DCFDA, Thermo Fisher Scientific, cat. no. D399) at 37°C for 30 min. Then, we collected the cells and washed them twice with 4°C PBS; PBS was used to resuspend the cells; FACScan Flow Cytometer (Beckman-Coulter) was immediately used to measure the fluorescence. The NADP/NADPH-Glo Kit (Promega, cat. no. G9081) was used to measure the intracellular levels of NADPH and for the total NADP measurement. The intracellular levels of GSH/GSSG was measured with a GSH/GSSG-Glo Assay kit (Promega, WI, USA). Annexin V-FITC and PI (4A Biotech Co. cat. no. FXP018) were used for cell apoptosis analysis with a flow cytometer. The cell cycle was analyzed by PI/RNase staining buffer (BD Pharmingen™) with a flow cytometer.

### MTHFD1L Promoter Luciferase Plasmids and Luciferase Reporter Assay

The dual fluorescent reporter gene plasmids with MTHFD1L promoter was conducted (Primers used to amplify the MTHFD1L promoter region: 5′- CTGGTACAGCTTACCAAAC-3′, 5′- TTCTCAGGGGACACGGAGCT-3′).We inserted the MTHFD1L promoter fragment to pGL4.10 - Luc2 between HindIII and XhoI sites in pGL4.10—Luc2 (Promega, AY738222) plasmid. We seeded CAL-27 cells and SCC-15 cells in 24-well plates and cultured with rapamycin for 24 h, then transfected with the MTHFD1L promoter luciferase plasmids. After 36 h we measured the luciferase activity by luciferase reporter assay kit (Promega Biotech Co., Ltd., Madison, WI, USA).

### Animal Experiments

We obtained 30 4-week-old female BALB/c nude mice from Vital River Laboratory Animal Technology Co., Ltd. (Beijing, China), we quarantined them for 1 week before the animal experiments in an animal laboratory center. We suspended the cells for the *in vivo* tumorigenesis study. MTHFD1L-SC or shMTHFD1L-1 and shMTHFD1L-2 (3 × 10^6^ cells, in PBS) were injected into BALB/c mice. We measured the volume of the tumors and the weight of the mice every 2 days for nearly 1 month. The mice were sacrificed at the end of the experiment. Tumors were excised, photographed, and processed for immunohistochemical analyses. All animal-associated experimental procedures were approved by the Animal Care and Use Committee of Sun Yat-sen University. We made every effort to reduce the suffering of the animals.

### Immunohistochemistry (IHC)

A total of 106 formalin-fixed paraffin-embedded (FFPE) TSCC tissue sections were provided by the Sun Yat-sen University Cancer Center. These sections were incubated with anti-MTHFD1L primary anti-bodies and secondary anti-bodies. Scoring for both the percentage and the intensity of positively stained tumor cells was performed by two independent pathologists under double-blind conditions.

### Statistical Analysis

Statistical analyses were performed using the SPSS statistical software package (version 24.0 SPSS Inc., Chicago, IL, USA) to evaluate the relationship between MTHFD1L expression and TSCC clinicopathological features. We used Kaplan-Meier curves and the log-rank test to analyze disease-free survival and overall survival. *P* < 0.05 was considered to be statistically significant.

## Results

### MTHFD1L Is Highly Expressed in TSCC

A Pyeon Multicancer study on the Oncomine database demonstrated that the MTHFD1L expression level was 4.50 times higher in TSCC tissues (15 samples) than in normal head and neck tissues (14 samples) and in cervix and uteri tissue (8 samples) (Figure 1A) (18). MTHFD1L was also found to be expressed at higher levels in head and neck cancer tissues than in normal tissues (*P* < 0.05) in several other studies (Figure 1B) (30–33).

To verify these findings, we analyzed the levels of MTHFD1L mRNA and protein expression separately in TSCC tissues (T) and adjacent non-carcinoma tissues (ANTs). The qRT-PCR results showed that the expression of MTHFD1L was significantly higher in the cancerous tissues than in the ANTs in 48 pairs of TSCC patients' fresh frozen tissues (*P* < 0.0001; Figure 1C). Western blot analysis also showed that expression of MTHFD1L was much more higher in the cancerous tissues than in the ANTs in 10 pairs of TSCC patients' fresh frozen tissues (Figure 1D).

### The Clinicopathological Features of MTHFD1L in TSCC

**Figure 2:**
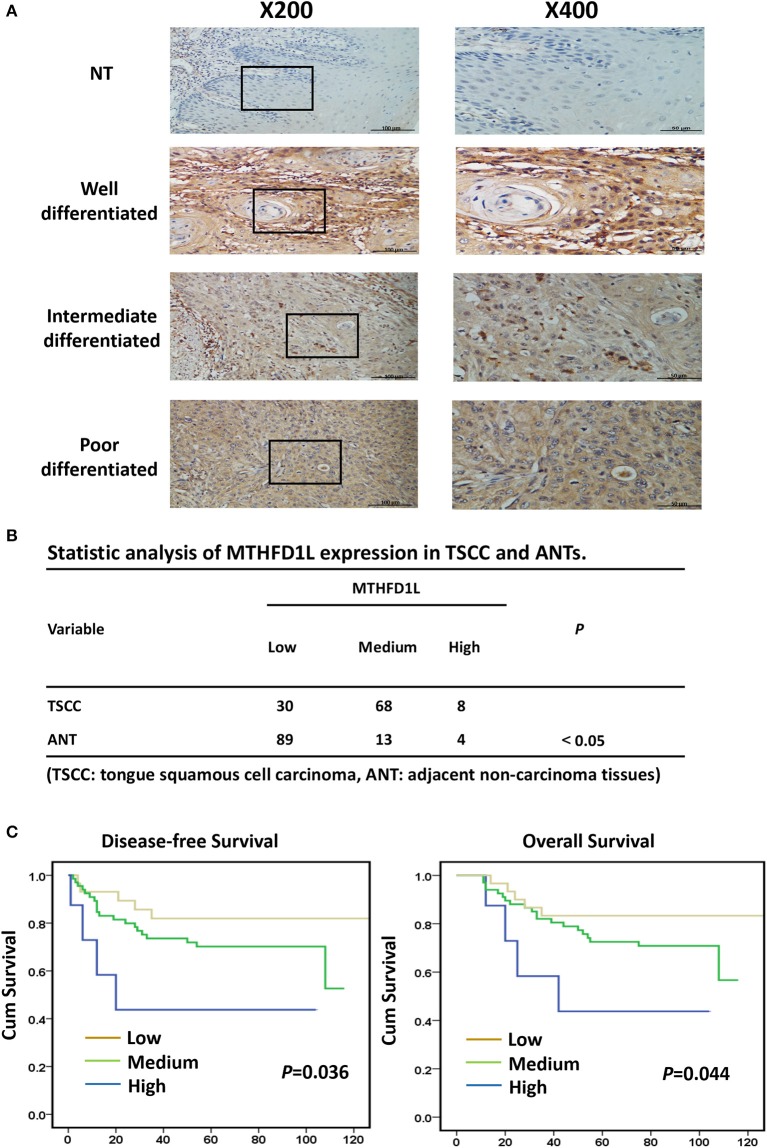
The clinicopathological features of MTHFD1L in TSCC. **(A)** Immunohistochemical results demonstrated the MTHFD1L expression in TSCC tissues and compared ANTs (*n* = 106). **(B)** Statistic analysis of MTHFD1L expression in TSCC and ANTs. **(C)** 5-year overall survival or disease-free survival for TSCC patients with low, medium, high expression of MTHFD1L (Kaplan-Meier analysis with the log-rank test; *n* = 106).

In order to further explore the expression of MTHFD1L in TSCC tissue specimens and its correlation with clinicopathological characteristics in TSCC, we performed immunohistochemical analysis of 106 paraffin-embedded TSCC tissues with matched ANTs to evaluate MTHFD1L expression levels in TSCC. In our study, the immunohistochemical results demonstrated that the MTHFD1L protein was expressed at much higher levels in the tumor tissues than in the matched ANTs (Figure 2A). Statistical analysis of MTHFD1L expression in TSCC and ANTs was showed in Figure 2B. Then, we analyzed the potential correlation between the associated clinicopathological features of TSCC patient tissues and MTHFD1L protein expression.

The level of MTHFD1L expression and the clinicopathological features of TSCC patients are described in Table 1. The expression level of MTHFD1L was correlated with age staging (*P* = 0.024), T classification (*P* < 0.001), N classification (*P* = 0.003), clinical TNM stage (*P* = 0.001), but not with relapse (*P* = 0.622), gender (*P* = 0.232), differentiation state (*P* = 0.476), and M classification (*P* = 0.754; Table 1). These results indicated that high MTHFD1L expression was associated with advanced TNM stage in TSCC.

By comparing the overall survival time(OS) and disease-free survival time(DFS) with the MTHFD1L expression (low, medium, high) of TSCC patients, we found that high MTHFD1L expression was associated with decreased disease-free survival time (*P* = 0.036) and with poor prognosis (*P* = 0.044) in Figure 2C. We further performed the Kaplan-Meier survival analysis in the MTHFD1L expression subgroup. The results indicated that patients with high expression of MTHFD1L had shorter OS and DFS than patients with low expression of MTHFD1L. (*P* = 0.017 for OS, *P* = 0.014 for DFS; Supplementary Table 1). These results indicated that expression of MTHFD1L was associated with shorter survival of TSCC patients.

### MTHFD1L Is Essential for TSCC Cell Proliferation and Cell Cycling

**Figure 3:**
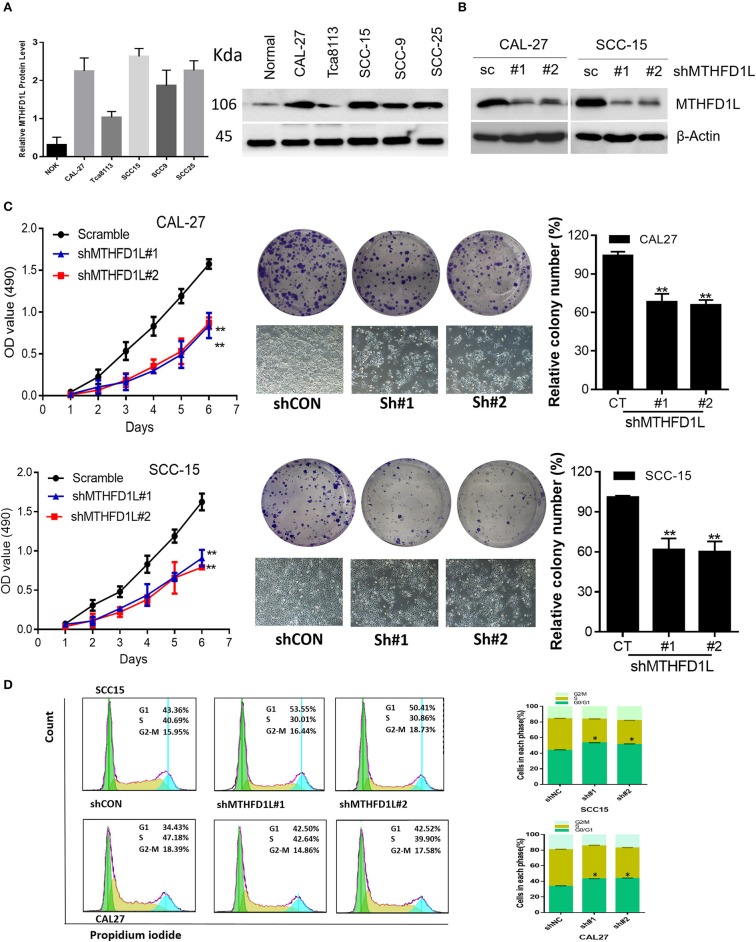
MTHFD1L is essential for TSCC cell proliferation and cell cycle progression. **(A)** Western blotting analysis of MTHFD1L protein levels in TSCC cell lines (CAL-27, Tca8113, SCC-15, SCC-9, SCC-25) and human normal tongue epithelial cell line (NOK). **(B)** Immunoblotting evaluating the knockdown efficiency of MTHFD1L with 2 unique shRNAs (#1, #2) in CAL-27 and SCC-15 cells. **(C)** Cell proliferation and clonogenicity were suppressed by down-regulating MTHFD1L in CAL-27 and SCC-15 cells (^**^P < 0.01). **(D)** Representative pictures of flow cytometry analyses of propidium iodide staining in MTHFD1L knockdown cells and negative control cells (CAL-27 and SCC-15 cell line) and cells in each phase (G2/M, S, G0/G1), with knockdown cells mainly remaining in the G1 phase (^*^P < 0.05).

Furthermore, we examined the expression of MTHFD1L at the protein level in a human normal tongue epithelial cell line (NOK) and in TSCC cell lines (CAL-27, Tca8113, SCC-15, SCC-9, SCC-25) by Western blotting (Figure 3A). Among the cell lines examined, the expression of MTHFD1L was higher in the TSCC cell lines than in the NOK cell lines.

MTHFD1L plays an important role in the folate cycle, and folate metabolism can affect nucleic acid formation, then influencing cell proliferation (22). Thus, to clearly identify the functions of MTHFD1L in the TSCC cell lines, we knocked down MTHFD1L expression in the CAL-27 and SCC-15 cell lines (Figure 3B). As expected, TSCC cell viability and colony formation were inhibited in the MTHFD1L knockdown cell line. After knocking down MTHFD1L, the cell proliferation rate, as well as the relative colony numbers, were significantly lower than those of the negative control cells (Figure 3C). We also observed that the knockdown of MTHFD1L affected the cell cycle, as the cell cycle was delayed in MTHFD1L knockdown cell lines compared with the negative control cells, with knockdown cells mainly remaining in the G1 phase (Figure 3D, Right). This result is statistically significant (Figure 3D, Left).

### MTHFD1L Inhibits TSCC Cell Apoptosis via Antioxidant Activity

**Figure 4:**
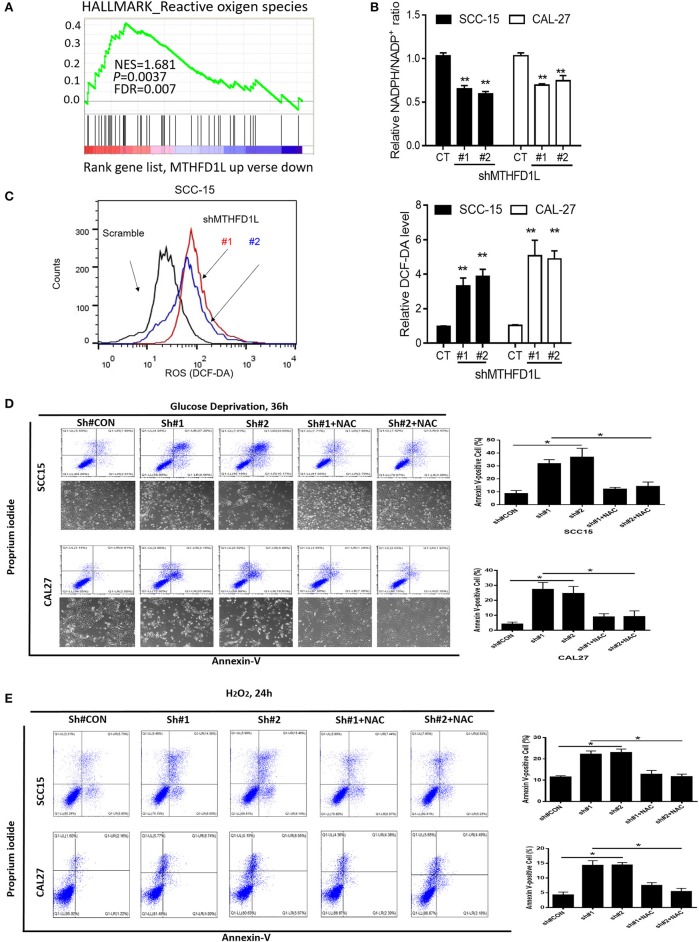
MTHFD1L inhibited TSCC cell apoptosis via anti-oxidant. **(A)** Gene set enrichment analysis (GSEA) indicated that the expression of MTHFD1L is related to reactive oxygen species (ROS)-related gene [results analyzed from The Cancer Genome Atlas (TCGA) TSCC database]. **(B)** Representative histograms of relative NADPH/NADP+ levels in MTHFD1L knockdown TSCC cells compared with negative control cells (CAL-27 and SCC-15 cells) (^**^P < 0.01). **(C)** Representative histograms and levels of intracellular ROS in MTHFD1L knockdown TSCC cells compared with negative control cells (CAL-27 and SCC-15 cells), as detected by the fluorescent probe 20,70-dichlorodihydrofluorescein diacetate (DCF-DA) (^**^P < 0.01). **(D,E)** Cell apoptosis was measured by Annexin-V/PI assays in MTHFD1L knockdown TSCC cells and negative control cells cultured in glucose deprivation medium for 36 h or under hypoxia for 72 h, histograms indicated subpopulation of cells positive for Annexin V/PI [with or without 5 mM N-acetyl-L-cysteine (NAC)] (^*^P < 0.05).

We performed gene set enrichment analysis (GSEA), and the signature of ROS-related genes was more abundant in tumors with high expression of MTHFD1L (normalized enrichment score = 1.681, *P* = 0.0037; Figure 4A) (The list of the detected enriched pathways was presented in Supplementary Table 2), revealing MTHFD1L acts important roles in redox homeostasis. MTHFD1L plays critical roles in folate cycle maintenance, which is an important source of NADPH (18). Thus, we knocked the MTHFD1L expression in the CAL-27 and SCC-15 cell lines, finding that the NADPH/NADP^+^ ratio was reduced (as well as GSH/GSSG, Supplementary Figure 1) and that the ROS levels increased (Figures 4A–C). We also found that the MTHFD2 expression reduced after MTHFD1L knockdown (Supplementary Figure 2). To analyze apoptosis, we generated an oxidative stress environment under glucose deprivation and added H_2_O_2_ separately. We observed that the cell death rate of MTHFD1L-knockdown cells was higher than that of the negative control cells. Interestingly, whether glucose deprivation or added H_2_O_2_ induced cell death could be rescued by adding the anti-oxidant N-acetyl-L-cysteine (NAC) (*P* < 0.01; Figures 4D,E). These data demonstrate that MTHFD1L is essential for the maintenance of redox homeostasis and facilitates TSCC cell survival during oxidative stress.

### MTHFD1L Promotes TSCC Tumorigenesis *in vivo*

**Figure 5:**
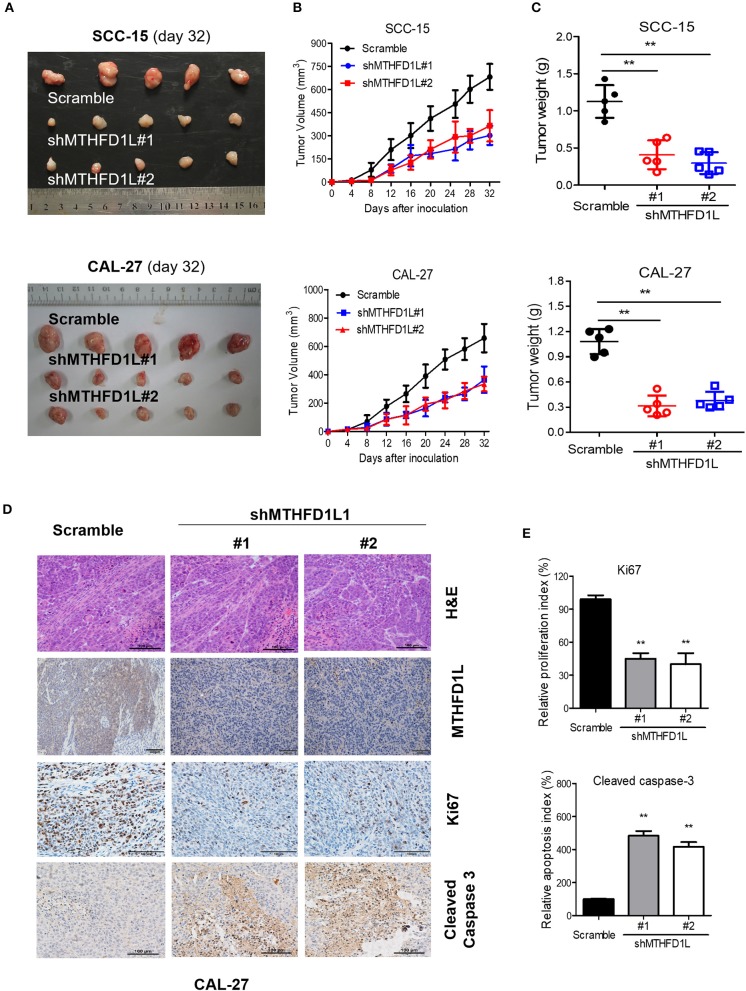
MTHFD1L promoted TSCC tumorigenesis *in vivo*. **(A)** MTHFD1L knockdown suppressed TSCC cells (CAL-27 and SCC-15 cells) growth in a mouse xenograft. Images of the thyroid cancer cell tumor xenograft from each mouse (*n* = 5 mice/group). **(B,C)** Tumor volumes and weights were analyzed. ^*^P < 0.05. **(D)** The expression of MTHFD1L, Ki67 and Cleaved Caspase 3 in tumor tissues was analyzed by IHC staining. **(E)** Quantification of the proliferation index (Ki67 staining) and apoptosis index (Cleaved caspase-3 staining) in tumor sections.

We then performed cell-based xenograft experiments to evaluate the relationship between MTHFD1L expression and how it influenced tumorigenic ability in nude mice. As expected, the MTHFD1L knockdown group had slower tumor growth and lower tumor weight than the negative control group (*P* < 001, for control vs. knockdown groups; Figures 5A–C). We observed that in the CAL-27 MTHFD1L knockdown tumor biopsies. Immunohistochemistry indicated that there were reduced cell proliferation indices based on Ki67 and increased cell-apoptosis-associated indices based on cleaved caspase-3 (*P* < 0.05 for control vs. knockdown groups; Figures 5D,E). These results highlight the crucial roles of MTHFD1L in TSCC tumorigenesis.

### Signaling Pathway of MTHFD1L Expression in TSCC

**Figure 6:**
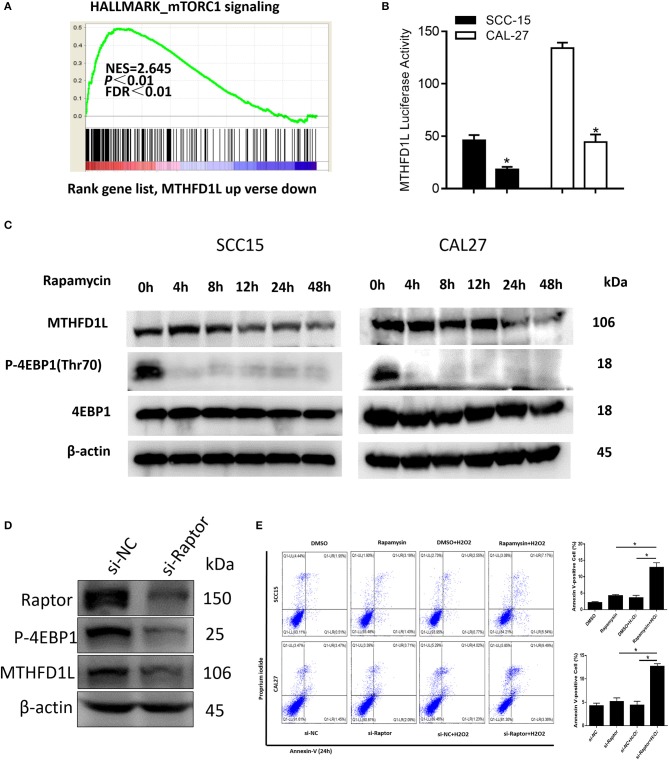
The mTORC1-4EBP1-eIF4E axis may affect the expression of MTHFD1L in TSCC. **(A)** Gene set enrichment score and distribution of mTORC1 signaling genes along the rank of MTHFD1L up vs. down available from The Cancer Genome Atlas TSCC database. **(B)** Relative MTHFD1L promoter activity in rapamycin treated TSCC cells (100 nM, 24 h) (^*^P < 0.05). **(C)** The expression of MTHFD1L in SCC-15/CAL-27 cell line after rapamycin treated at different time (50 nM). **(D)** The expression of MTHFD1L in CAL-27 cell line after knockdown of raptor. **(E)** Cell apoptosis was detected after the treatment of rapamycin or knockdown of raptor expression in TSCC cells (with or without H_2_O_2_, 100 μM, 48 h) (^*^P < 0.05).

In summary, we have revealed that MTHFD1L promotes the development of TSCC by regulating the redox homeostasis. To further analyze the molecular regulation mechanism that may be involved, GSEA analysis indicated that the MTHFD1L expression is positively correlated with mTORC1 signaling pathway in TSCC (normalized enrichment score = 2.645, *P* < 0.01, Figure 6A, the list of the detected enriched pathways was presented in Supplementary Table 2). The mTORC1 signaling pathway is a typical signaling pathway involved in protein synthesis and cell proliferation (34). In addition, mTORC1 can activate cells through metabolic reprogramming, which makes cells dependent on glucose and glutamine uptake, especially in metabolic stress environments (35).

In order to verify whether mTORC1 affects the expression of MTHFD1L in TSCC, we treated TSCC cells with mTORC1 specific inhibitor rapamycin, and found that the activity of MTHFD1L promoter activity decreased significantly (Figure 6B).

In addition, rapamycin can significantly inhibit the phosphorylation of downstream substrate 4EBP1 (Figure 6C). Phosphorylated 4EBP1 can promote the release of eIF4E, and phosphorylation of Thr70 is most important for the release of eIF4E (36). eIF4E is the most important eukaryotic translation initiation factor, which is the most effective rate-limiting regulator of mRNA translation (37), and the decrease in p-4EBP1 is accompanied by a decrease in the expression of MTHFD1L (Figure 6C). Then, we also knocked down the expression of raptor, an important component of mTORC1 activity, in the CAL-27 cell line, and found that the phosphorylation of the downstream substrate 4EBP1 was inhibited (Figure 6D). Conversely, we knocked down the expression of MTHFD1L and found that other readouts of mTORC1 activity such as p-S6RP didn't change their expression (Supplementary Figure 2). In consequence, we predict that the mTORC1-4EBP1-eIF4E axis may affect the expression of MTHFD1L, which indicating that mTORC1 has a positive regulation of MTHFD1L in TSCC.

At the same time, we found that there was no significant change in cell apoptosis after treatment of TSCC cells with rapamycin or knockdown of raptor expression in TSCC cells. Interestingly, apoptosis was increased after added H_2_O_2_ (Figure 6E).

## Discussion

The folate cycle is associated with the maintenance of epigenetic modifications, nucleotide synthesis and anti-oxidant production, these biological events are always associated with tumorigenesis and tumor development. However, we do not know much about its effects on TSCC, hence this topic attracted our interest. MTHFD1L is a crucial folate cycle component. In this study, MTHFD1L was highly expressed in TSCC tissues compared with adjacent non-carcinoma tissues (ANTs), and its high expression was also associated with advanced clinical TNM stage and poor prognosis, demonstrating that MTHFD1L could be a prognostic indicator in TSCC. To determine the function of MTHFD1L in TSCC cell lines, we established MTHFD1L knockdown cell lines that effectively decreased cell proliferation rates and caused a cell cycle delay. All of these effects were related to NADPH reduction and ROS accumulation, as well as cell apoptosis and TSCC growth inhibition *in vivo*. In our next study, we also want to figure out the regulation mechanism of MTHFD1L expression in TSCC lymphatic metastasis.

It is well-known that MTHFD1L is overexpressed in multiple solid cancers, demonstrating that the folate cycle plays an important role in tumor metabolism and in accelerating tumor growth. Many conventional chemotherapies and radiotherapies eradicate cancer cells through ROS induction (26). These findings will inspire us to find more effective ways to treat cancer, such as the sensitization of existing cancer therapies using interference with folate circulation.

Additionally, the regulatory mechanism of MTHFD1L in TSCC should be studied in more depth. Previous studies have demonstrated that MTHFD1L is transcriptionally regulated by the transcription factor nuclear factor (erythroid-derived 2)-like 2 (NRF2) (18), which acts as the central regulator for redox homeostasis (38). The KEAP1/NRF2 pathway is the key pathway that provides defense against oxidative stress, and it has been indicated to be the most frequently mutated pathway in human HCC (39). Whether this pathway plays a role in TSCC metabolism will be explored further in our next study.

The epigenetic regulation of metabolism-related genes has also been reported in several studies that have attracted our attention. MTHFD1L silencing reduced proliferation and enhanced the apoptosis of non-small cell lung cancer by suppressing DNA methylation (40). MTHFD1L supports the Flow of Mitochondrial One-carbon Units into the Methyl Cycle in Embryos as well (22). These reports provide support for our future research directions and further confirm the possibility that metabolic genes could serve as targets for tumor treatment. In the present study, the MTHFD1L expression could be reduced by mTORC1 inhibition and knockdown of raptor. We further found that mTORC1 facilitated the expression of MTHFD1L by promoting the phosphorylation of 4EBP1 to release eIF4E, thereby affecting the development of TSCC.

## Conclusion

The expression of MTHFD1L accelerates the tumorigenesis in TSCC. Inhibition of the expression of MTHFD1L may become an actionable and effective therapeutic target in TSCC. This finding may also provide inspiration for us to study more about tumor metabolism in head and neck cancer.

## Data Availability Statement

All of the authors declare that all the data of this study are available in this article.

## Ethics Statement

TSCC tissues were collected from patients who first diagnosed in Sun Yat-sen University Cancer Center and underwent surgical resection in the Head and Neck Surgery Department (SYSUCC, Guangzhou, China). All patients signed consent letters, and any manipulation of the tissues was approved by the Ethics Committee of Sun Yat-sen University. All animal procedures were performed in accordance with the guidelines of the Institutional Animal Care and Use Committee and the guidelines of Guangzhou Medical University and Sun Yat-sen University.

## Author Contributions

HL, AY, and XF designed the study. TT, FY, and CW performed the *in vitro* and animal experiments. HL, XF, TT, and FY analyzed the data and wrote the manuscript. All of the authors read and approved the final manuscript.

### Conflict of Interest

The authors declare that the research was conducted in the absence of any commercial or financial relationships that could be construed as a potential conflict of interest.

## Abbreviations

TSCC: tongue squamous cell carcinoma
MTHFD1L: methylenetetrahydrofolate dehydrogenase 1-like
NADPH: nicotinamide adenine dinucleotide phosphate
ROS: reactive oxygen species
SCC: squamous cell carcinoma
MTHFD2: methylene tetrahydrofolate dehydrogenase 2
THF: tetrahydrofolate
WHO: World Health Organization
AJCC: American Joint Committee on Cancer
SD: standard deviation
FBS: fatal bovine serum
PVDF: polyvinylidene fluoride
RT-PCR: Reverse Transcription-Polymerase Chain Reaction
RNA: RibonucleicAcid
FFPE: formalin-fixed paraffin-embedded
ANT: adjacent non-carcinoma tissue
IHC: immunohistochemical
OS: overall survival
DFS: disease-free survival.
