# Supplementary material for: MTHFD1L-Mediated Redox Homeostasis Promotes Tumor Progression in Tongue Squamous Cell Carcinoma
Source: Front Oncol. 2019 Dec 5;9:1278. doi: 10.3389/fonc.2019.01278 (PMC6906156; doi:10.3389/fonc.2019.01278)
Supplement: Supplementary file 1 [file Table_1.docx]

**Supplementary table 1: Log Rank Pairwise comparisons in MTHFD1L expression subgroup.**

| **MTHFD1L**  **expression** | **Low vs Medium** | |  | **Medium vs High** | |  | **Low vs High** | | |
| --- | --- | --- | --- | --- | --- | --- | --- | --- | --- |
|  | **Chi-square** | **Sig.** |  | **Chi-square** | **Sig.** |  | **Chi-square** | | **Sig.** |
| **OS** | **2.161** | **0.142** |  | **2.916** | **0.088** |  | **5.713** | | **0.017** |
| **DFS** | **2.079** | **0.149** |  | **3.279** | **0.070** |  | | **5.985** | **0.014** |

**(OS: overall survival; DFS: disease free survival.)**
