# Supplementary figures and images for: MTHFD1L-Mediated Redox Homeostasis Promotes Tumor Progression in Tongue Squamous Cell Carcinoma
Source: Front Oncol. 2019 Dec 5;9:1278. doi: 10.3389/fonc.2019.01278 (PMC6906156; doi:10.3389/fonc.2019.01278)

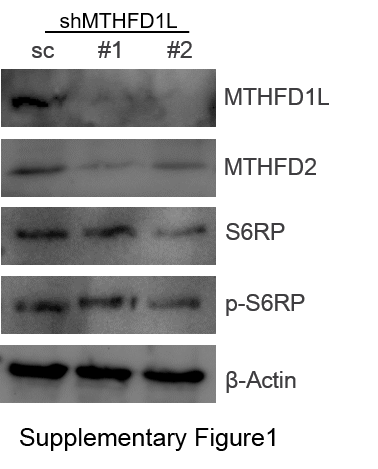

Supplement: Supplementary file 3 [file Image_1.tif]

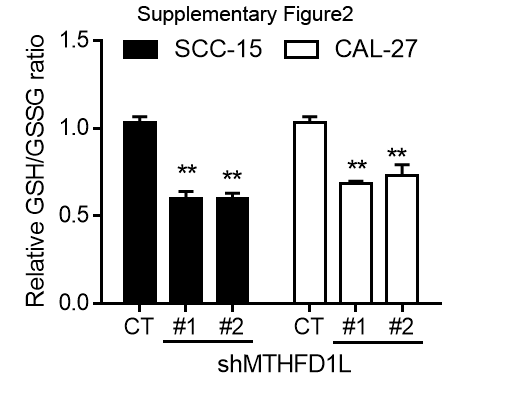

Supplement: Supplementary file 4 [file Image_2.tif]
